# Comparative analysis of the tonsillar microbiota in IgA nephropathy and other glomerular diseases

**DOI:** 10.1038/s41598-020-73035-x

**Published:** 2020-10-01

**Authors:** Ji In Park, Tae-Yoon Kim, Bumjo Oh, Hyunjeong Cho, Ji Eun Kim, Seong Ho Yoo, Jung Pyo Lee, Yon Su Kim, Jongsik Chun, Bong-Soo Kim, Hajeong Lee

**Affiliations:** 1 Department of Internal Medicine, Kangwon National University Hospital, Kangwon National University School of Medicine, Chuncheon, Republic of Korea; 2 Department of Life Science, Multidisciplinary Genome Institute, Hallym University, 1 Hallymdaehak-gil, Chuncheon, Gangwon-do Republic of Korea; 3 Illumina, Inc, Seoul, Republic of Korea; 4 Department of Family Medicine, SMG-SNU Boramae Medical Center, Seoul, Republic of Korea; 5 Department of Internal Medicine, Chungbuk National University Hospital, Cheongju, Republic of Korea; 6 Department of Internal Medicine, Seoul National University Hospital, 101 Daehak-ro, Jongno-gu, Seoul, Republic of Korea; 7 Department of Internal Medicine, Korea University Guro Hospital, Seoul, Republic of Korea; 8 Department of Forensic Medicine and Institute of Forensic Medicine, Seoul National University College of Medicine, Seoul, Republic of Korea; 9 Department of Internal Medicine, Seoul National University Boramae Medical Center, Seoul, Republic of Korea; 10 School of Biological Sciences, Seoul National University, Seoul, Republic of Korea

**Keywords:** Microbiology, Nephrology

## Abstract

Immunoglobulin A nephropathy (IgAN) involves repeated events of gross haematuria with concurrent upper airway infections. The mucosal immune system, especially the tonsil, is considered the initial site of inflammation, although the role of the tonsillar microbiota has not been established in IgAN. In this study, we compared the tonsillar microbiota of patients with IgAN (n = 21) and other glomerular diseases (n = 36) as well as, healthy controls (n = 23) from three medical centres in Korea. The microbiota was analysed from tonsil swabs using the Illumina MiSeq system based on 16S rRNA gene. Tonsillar bacterial diversity was higher in IgAN than in other glomerular diseases, although it did not differ from that of healthy controls. Principal coordinates analysis revealed differences between the tonsillar microbiota of IgAN and both healthy and disease controls. The proportions of *Rahnella*, *Ruminococcus_g2*, and *Clostridium_g21* were significantly higher in patients with IgAN than in healthy controls (corrected p < 0.05). The relative abundances of several taxa were correlated with the estimated glomerular filtration rate, blood urea nitrogen, haemoglobin, and serum albumin levels. Based on our findings, tonsillar microbiota may be associated with clinical features and possible immunologic pathogenesis of IgAN.

## Introduction

Immunoglobulin A nephropathy (IgAN) is the most prevalent primary glomerulonephritis worldwide^1^. It usually affects young individuals and a significant proportion of cases eventually progress to kidney failure^2, 3^. The prevalence and genetic risk of IgAN are higher and the kidney prognosis is worse in Asians than in other populations^4, 5^. IgAN has been recognised as a mild glomerulonephritis initially, but a significant proportion of patients progresses to end-stage kidney disease and their mortality rate is higher than matched controls^6, 7^. Although the aetiology and pathogenesis remain unclear, mucosal immunity is associated with the development of IgAN. Patients with IgAN frequently suffer from kidney manifestations, such as gross haematuria, flank pain, or kidney swelling, and rarely develop acute kidney injuries simultaneously or immediately after upper respiratory or gastrointestinal illness^8^. Various factors may explain these clinical features. For example, they could be related to serum galactose-deficient IgA1 level, which is important in the pathogenesis of the disease and is produced in mucosal tissues. The mucosal synthesis of galactose-deficient IgA1 is influenced by the innate immune system via toll-like receptors^9^. Additionally, the gut microbiota is a critical factor for the production of IgA in the intestinal mucosa of a mouse model^10^. Significant loci for IgAN are associated with the maintenance of the mucosal barrier and response to mucosal pathogens based on a genome-wide association study^11^.

The tonsil is a central site for antigen processing in the mucosal immune system and the first defensive organ against gastrointestinal entry. A common clinical manifestation of IgAN is macroscopic haematuria, which often coincides with tonsillitis^8, 12^. Accordingly, the tonsil is considered the initial site of inflammation, and tonsillectomy has well-established beneficial effects in patients with IgAN suffering from recurrent tonsillitis^13, 14^, either alone or in combination with steroid therapy^14–18^. Furthermore, several bacterial antigens are known to induce IgAN^19–22^.

The microbiota signature in the tonsils of patients with IgAN has not been fully elucidated. A recent study of the tonsillar microbiota did not show any difference among IgAN, recurrent tonsillitis, and tonsillar hypertrophy^23^. However, this previous study was based on single-centre data, and urinalysis data were lacking for paediatric patients in the control group, making it impossible to exclude hidden glomerular diseases. In this study, we characterised the tonsillar microbiota in patients with IgAN in comparison with that in healthy controls—consisting of live kidney donors without evidence of kidney disease—and disease controls with biopsy-proven diabetic nephropathy (DN) and membranous nephropathy (MN) in multiple centres. Our findings extend our understanding of the role of the tonsillar microbiota in the pathogenesis of IgAN.

## Results

### Clinical characteristics of subjects

A total of 80 subjects were included, including 21 patients with IgAN, 21 patients with MN, 15 patients with DN, and 23 healthy subjects. Clinical characteristics are summarised in Table 1. The mean age was lower in the healthy control group (32.8 ± 5.8 years) than in the disease groups (53.2 ± 14.4 years, p < 0.001). Hypertension was more prevalent in all disease groups than in healthy controls (p < 0.001). The levels of plasma haemoglobin, serum albumin, and serum calcium were lower in IgAN group than in healthy controls (p < 0.001). Patients with MN showed features of nephrotic syndrome, including hypercholesterolaemia, hypoalbuminaemia, and large amounts of proteinuria. Kidney function, as evaluated by the estimated glomerular filtration rate (eGFR), was lowest in DN, followed by IgAN. Proteinuria and haematuria were common in the disease controls and IgAN groups. The Oxford classification of the kidney pathology was available for 18 of 21 patients with IgAN and varied from minimal to severe (Table 2).

**Table 1:** Baseline characteristics of subjects.

	Healthy control (N = 23)	IgA nephropathy (N = 21)	Membranous nephropathy (N = 21)	Diabetic nephropathy (N = 15)	p-value
Age (years)	32.8 ± 5.8	47.8 ± 18.1	56.2 ± 11.0	56.5 ± 11.0	< 0.001
Male [N (%)]	15 (65.2)	11 (52.4)	13 (61.9)	12 (80.0)	0.401
Body mass index (kg/m^2^)	24.2 ± 4.1	25.1 ± 2.7	26.2 ± 5.9	25.6 ± 4.1	0.498
SBP (mmHg)	119.8 ± 14.9	127.8 ± 20.4	128.7 ± 17.0	134.4 ± 25.9	0.142
DBP (mmHg)	80.7 ± 12.5	81.1 ± 16.2	79.3 ± 11.7	78.9 ± 16.1	0.961
Smoking [N (%)]	4(17.4)	7 (33.3)	3 (14.3)	1 (7.1)	0.219
Hypertension [N (%)]	0 (0.0)	11 (52.4)	9 (42.9)	10 (66.7)	< 0.001
Diabetes mellitus [N (%)]	0 (0.0)	4 (19.0)	2 (9.5)	15 (100.0)	< 0.001
Haemoglobin (g/dL)	15.0 ± 1.6	13.0 ± 2.5	12.6 ± 1.7	10.4 ± 1.6	< 0.001
Glucose (mg/dL)	92.1 ± 9.1	117.7 ± 35.8	113.4 ± 24.2	135.5 ± 95.0	0.047
Total cholesterol (mg/dL)	196.3 ± 39.8	205.4 ± 77.9	249.1 ± 81.0	177.9 ± 45.4	0.009
Albumin (g/dL)	4.4 ± 0.2	3.5 ± 0.8	2.8 ± 0.7	3.5 ± 0.7	< 0.001
Calcium (mg/dL)	9.2 ± 0.4	8.6 ± 0.8	8.2 ± 0.6	8.6 ± 0.6	< 0.001
Phosphorus (mg/dL)	3.4 ± 0.2	3.6 ± 0.8	3.6 ± 0.5	3.8 ± 0.9	0.238
Uric acid (mg/dL)	5.7 ± 1.6	6.4 ± 1.7	6.2 ± 1.8	7.3 ± 2.9	0.116
Blood urea nitrogen (mg/dL)	11.1 ± 2.6	23.5 ± 19.1	14.8 ± 3.5	31.0 ± 15.3	< 0.001
Creatinine (mg/dL)	0.9 ± 0.1	1.7 ± 1.8	0.8 ± 0.2	2.2 ± 1.7	0.001
eGFR (mL/min/1.73 m^2^)	107.7 ± 12.8	69.0 ± 37.4	92.0 ± 15.6	45.6 ± 23.8	< 0.001
**Proteinuria [N (%)]**					< 0.001
Negative ~ trace	23 (100.0)	3 (14.3)	0 (0.0)	1 (7.1)	
1 + ~ 2 +	0 (0.0)	4 (19.0)	2 (9.5)	3 (21.4)	
3 + ~ 4 +	0 (0.0)	14 (66.7)	19 (90.5)	10 (71.4)	
**Haematuria [N (%)]**					< 0.001
< 1/HPF	13 (56.5)	0 (0.0)	1 (4.8)	4 (29.6)	
1 ~ 4/HPF	10 (43.5)	3 (14.3)	8 (38.1)	6 (42.9)	
> 5/HPF	0 (0.0)	18 (85.7)	12 (57.1)	4 (28.6)	
Urine protein to creatinine ratio (mg/mg)	–	3.7 ± 2.9	6.2 ± 3.8	5.9 ± 4.7	0.081

*SBP* systolic blood pressure, *DBP* diastolic blood pressure, eGFR; estimated glomerular filtration rate, HPF; high-power field. Data are presented as mean ± standard deviation or number (percentage); the p-value for comparison of all four groups.

**Table 2:** Distribution of histopathological changes in patients with IgA nephropathy according to the Oxford classification.

	Frequency (Total N = 18)	Percentage (%)
**Mesangial hypercellularity**		
M0	8	44.4
M1	10	55.6
**Endocapillary hypercellularity**		
E0	14	77.8
E1	4	22.2
**Segmental glomerulosclerosis**		
S0	9	50
S1	9	50
**Tubular atrophy/interstitial fibrosis**		
T0	11	61.1
T1	6	33.3
T2	1	5.6
**Crescent**		
C0	11	61.1
C1	6	33.3
C2	1	5.6

### Diversity and phylum composition of the tonsillar microbiota

**Figure 1:**
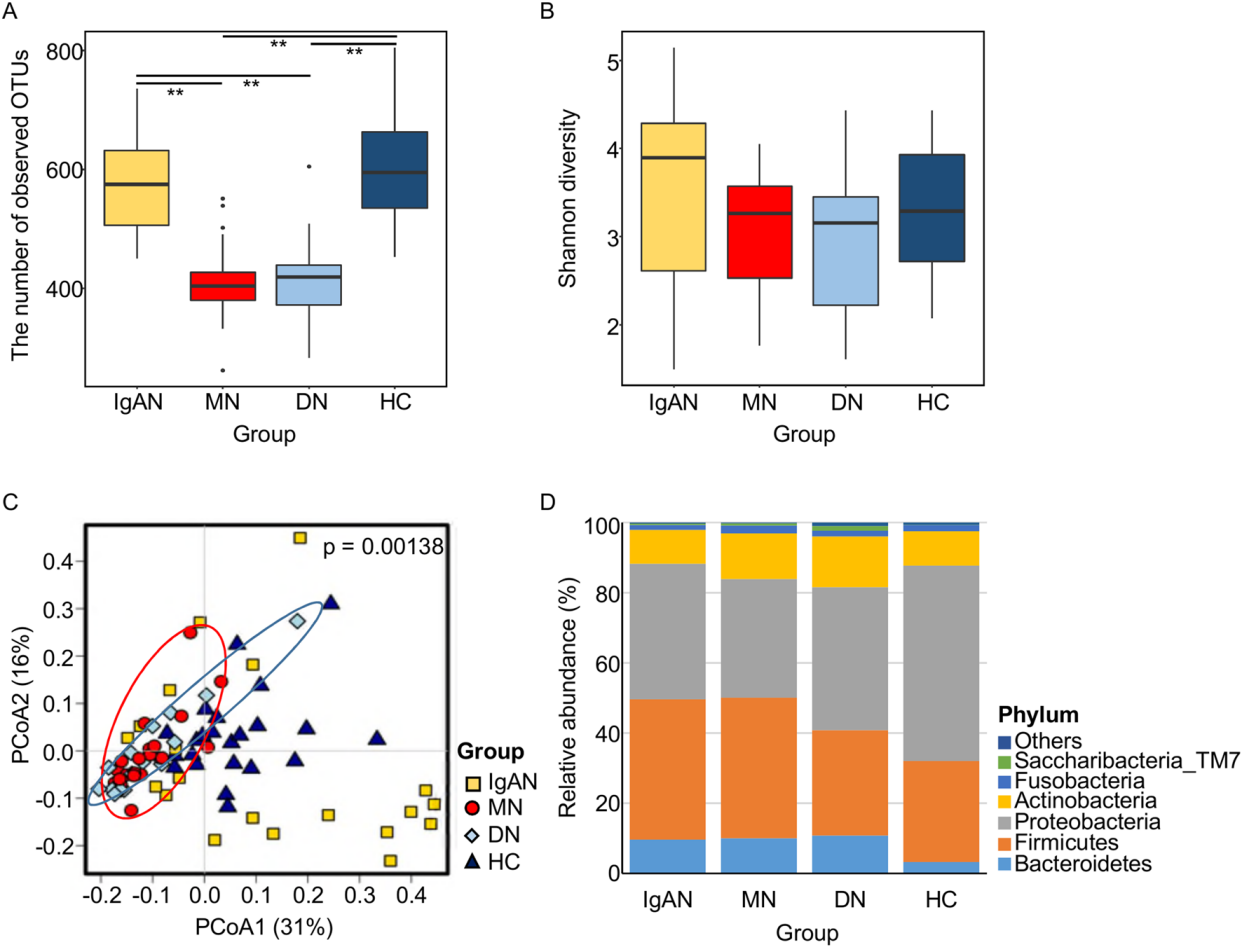
Comparison of diversity and phylum composition of the tonsillar microbiota among groups. (**A**) OTU counts were compared after the normalisation of the read number for each sample. (**B**) Shannon diversity indices were compared. (**C**) The principal coordinated analysis plot of tonsillar microbiota based on Bray–Curtis distances. Significance was estimated by permutation tests. (**D**) Comparison of the phylum composition among groups. Relative abundance is expressed as the mean value for each group. Comparisons were performed using Mann–Whitney U tests. (**p < 0.01). OTUs, operational taxonomic units.

A total of 3,443,894 sequence reads (average 43,048.7 reads per sample) were obtained from 80 tonsil swabs. The diversity indices after normalisation are summarised in Supplementary Table S1. Although the mean age differed significantly among groups (Table 1), there were no significant differences in bacterial diversity according to age in each group (p > 0.05; Supplementary Fig. S1). As shown in Fig. 1, we detected more operational taxonomic units (OTUs) in the IgAN and healthy control groups than in the MN and DN groups (p < 0.01). The compositions of the microbiota differed among groups in a Principal coordinates analysis (PcoA) based on Bray–Curtis distances (p < 0.01 by permutation tests; Fig. 1C). The microbiota of MN was more similar to that of DN than to those of the IgAN and healthy control groups. Individual variation was higher in the IgAN group than in the other groups. Firmicutes, Proteobacteria, Bacteroidetes, and Actinobacteria were the dominant phyla in all groups (Fig. 1D). The relative abundance of Proteobacteria (55.7% of the total microbiota) was higher in the healthy control than in the other groups (33.8% to 40.8%), whereas the proportions of Firmicutes and Bacteroidetes were higher in all disease groups than in healthy control groups. Although the average proportions of each phylum differed among groups, these differences were not statistically significant (corrected p > 0.05).

### Significantly different microbes among groups

**Figure 2:**
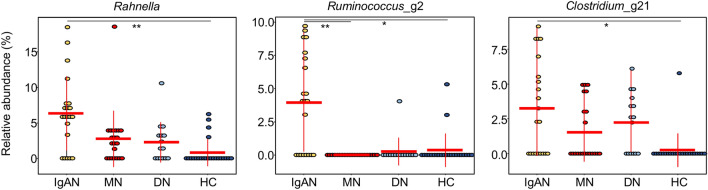
Genera with significant differences in abundance between patients with IgAN and the healthy control group. Significance was evaluated by Mann–Whitney tests and corrected by the Benjamini–Hochberg method (**corrected p < 0.01, *corrected p < 0.05).

To detect significantly different genera, frequently detected genera were compared among groups (Supplementary Fig. S2). Frequently detected genera were defined as genera that comprised > 0.5% (mean value) of the microbiota in more than 50% of the samples in each group. *Streptococcus*, *Pseudomonas*, *Neisseria*, and *Haemophilus* were the dominant genera (> 5% in each group) in all groups. The relative abundances of *Rahnella*, *Ruminococcus*_g2, and *Clostridium*_g21 were significantly higher in the IgAN group than in the healthy control group (corrected p < 0.05; Fig. 2). Other genera that differed significantly between healthy control and disease control groups or between IgAN and disease control groups are summarised in Supplementary Table S2. More genera differed between the MN and healthy control (17 genera) or IgAN groups (14 genera) than between the DN and healthy control (10 genera) or IgAN groups (1 genus) (corrected p < 0.05). *Tannerella*, *Eubacterium*_g10, *Faecalibacterium*, *Lachnoanaerobaculum*, uncultured *Veillonellaceae*, *Citrobacter, Acinetobacter,* and uncultured *Moraxellaceae* were commonly different between the healthy control and MN or DN groups. Conversely, there were no common genera between the IgAN and MN or DN groups.

### Correlation between genera and clinical features

**Figure 3:**
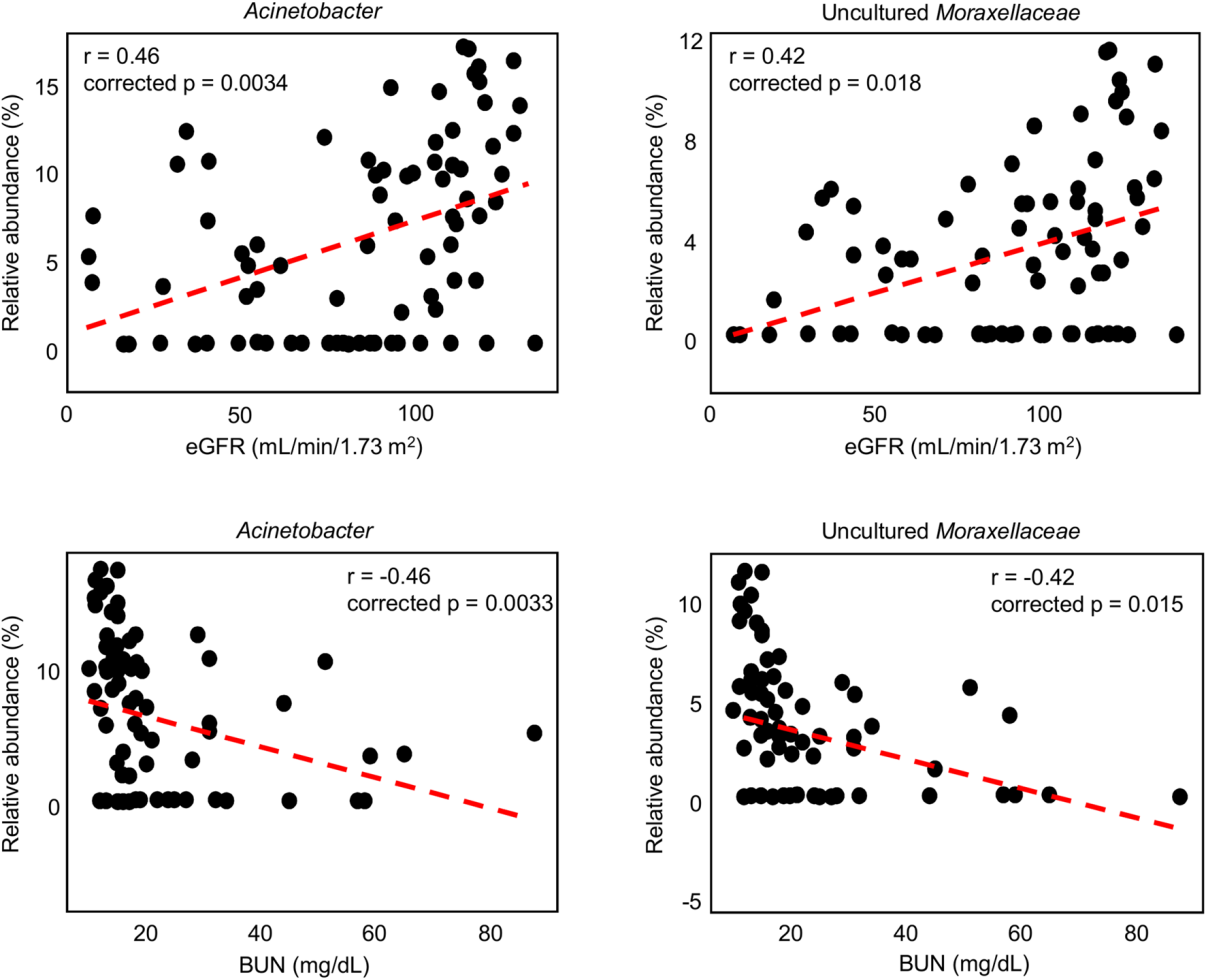
Correlations between the tonsillar microbiota and clinical features in all participants. Correlations of *Acinetobacter* and uncultured *Moraxellaceae* with eGFR and BUN were evaluated by corrected p-values. eGFR, estimated glomerular filtration rate; BUN, blood urea nitrogen.

We analysed the correlations between the relative abundances of genera and clinical features using Spearman’s rank correlation coefficients with corrected p-values. Four genera having significantly different abundance between the IgAN and healthy control were also correlated with clinical features (corrected p < 0.05). The relative abundances of two genera, *Acinetobacter* and uncultured *Moraxellaceae*, were correlated with kidney function represented by eGFR and blood urea nitrogen (BUN) (Fig. 3). In particular, they were positively correlated with eGFR values and negatively correlated with BUN values. In addition, *Acinetobacter*, uncultured *Moraxellaceae*, and *Enterobacter* were positively correlated with plasma haemoglobin values (Supplementary Fig. S3). *Acinetobacter*, uncultured *Moraxellaceae*, and *Delftia* were positively correlated with serum albumin values (Supplementary Fig. S3). Three genera with significant differences in abundance between the healthy control or IgAN and disease control groups were correlated with eGFR, albumin, BUN, and haemoglobin values (Supplementary Fig. S4). *Tannerella* was negatively correlated with eGFR values, and *Citrobacter* was positively correlated with albumin values. *Capnocytophaga* was positively correlated with BUN values but negatively correlated with haemoglobin values.

## Discussion

We detected genus-level differences in the microbiota of the tonsils of patients with IgAN, patients with other kidney diseases (MN and DN), and healthy controls. *Rahnella*, *Ruminococcus*_g2, and *Clostridium*_g21 were relatively abundant in the tonsils of patients with IgAN. In addition, the tonsillar microbiota was related to clinical features, especially kidney function. To our knowledge, this is the first comparative analysis of the tonsillar microbiota of patients with IgAN and other glomerular diseases.

The dominant phyla in tonsil samples were Proteobacteria, Firmicutes, Actinobacteria, and Bacteroidetes, consistent with previous findings (Fig. 1)^24^. Bacterial richness was higher in the IgAN and healthy control groups than in the MN and DN groups. In previous studies, *Haemophilus parainfluenzae* and *Staphylococcus aureus* have been identified as candidates for the induction of IgAN^19–22^. *Treponema* and *Campylobacter rectus* are also associated with the development and progression of IgAN^25^. *Prevotella*, *Porphyromonas*, and *Treponema* are more abundant in patients with IgAN than in patients with tonsillar hyperplasia^23^. However, in our study, *Haemophilus* was the dominant genus in all groups; *Staphylococcus*, *Treponema*, and *Campylobacter* were detected in all groups (Supplementary Fig. S2) and differences in relative abundances were not significant. We found that the relative abundances of *Rahnella*, *Ruminococcus_g2*, and *Clostridium_g21* were significantly higher in IgAN than in healthy controls (corrected p < 0.05; Fig. 2). These inconsistencies with previous studies have several potential explanations, including differences in sampling sites, methods, and the ethnicity of subjects. Differences in the composition of the microbiota between the tonsil surface and tissues have been observed^26^. Furthermore, most previous studies have focused on specific bacteria in the pathogenesis of IgAN. Our high-throughput sequencing approach provided an overview of the comprehensive bacterial microbiota. Differences between our results and those of a previous study using a high-throughput sequencing approach^23^ could be explained by differences in sample collection and study populations, which are known to effect the human microbiota^27, 28^.

Three bacteria showed higher abundances in the IgAN group than in the healthy control group. *Rahnella* sp. has been isolated from the blood, surgical wounds, urine, sputum, bronchial washings, tonsil, and stool^29^. This bacterium can cause bacteraemia from a kidney focus^30^ and infection in immunosuppressed individuals^31^. *Ruminococcus_g2* and *Clostridium_g21* were evaluated by a hierarchical clustering analysis of reference sequences in the EzTaxon-e database (https://www.ezbiocloud.net/) based on 16S rRNA genes. *Ruminococcus_g2* belongs to a cluster including *Ruminococcus bromii* and uncultured *Ruminococcus*. *R. bromii* has been detected in the microbiota of adenoiditis and tonsillitis^32^ and in the intestine of patients with HIV-1 infection^33^. *Clostridium_g21* belongs to a cluster including *Clostridium scindens* and uncultured *Clostridium*. *C. scindens* can covert glucocorticoids into androgens^34^ and is involved in resistance to *C. difficile* infection^35^. These three bacteria related to tonsillitis are especially abundant in the tonsils of patients with IgAN. They are therefore novel candidates for the pathogenesis of IgAN or for related differences in the immune system status.

We did not detect genera with significant differences in abundance between the MN and DN groups. We observed more genera differing between the healthy control and MN (17 genera) or DN groups (10) than between the healthy control and IgAN groups (6). The observed differences in the tonsillar microbiota among kidney diseases could be associated with the pathogenesis or progression of each disease. In particular, we detected greater differences between MN and healthy control or IgAN groups than between DN and the healthy control or IgAN groups. The development of DN involves chronic systemic inflammation, whereas primary MN is an autoimmune disease mainly mediated by antibodies to podocyte antigens, the M-type phospholipase A2 receptor and thrombospondin type 1 domain-containing 7A^36^. The tonsil has immunological functions, and the tonsillar microbiota may be more closely related to pathogenesis of MN than to the pathogenesis of DN. Differentially abundant genera in the tonsillar microbiota of each kidney disease are candidates for further functional studies.

We detected correlations between the relative abundances of genera and clinical features of kidney functions (Fig. 3 and S3). Anaemia is a well-known clinical feature of acute kidney injury or chronic kidney disease^37, 38^. Hypoalbuminaemia is common in advanced chronic kidney disease including including end-stage kidney disease^39^ and is associated with high mortality in acute kidney injury and chronic kidney disease^40^. In our study, *Acinetobacter* and uncultured *Moraxellaceae* were related to better kidney function and higher levels of plasma haemoglobin and serum albumin in all kidney diseases. The similar patterns for the relationships between these parameters and specific bacteria provide a basis for further clinical studies, particularly given the lack of research on the role of tonsillar microbiota in kidney diseases.

In addition, three genera with significant differences in relative abundance between healthy control or IgAN and disease control groups were correlated with eGFR, serum albumin, BUN, and plasma haemoglobin (Supplementary Table S2 and Supplementary Fig. S4). *Tannerella,* found in the oral cavity and tonsilloliths, is associated with the production of volatile sulphur compounds and periodontitis^41, 42^, which is linked to chronic kidney disease^43^. The correlation between a high abundance of *Tannerella* and decreased kidney function (as evaluated by eGFR) might be related to periodontitis caused by this pathogen. *Capnocytophaga* is a core component of the microbiota in the oral cavity and palatine tonsil of HIV-infected individuals^44^. *C. ochracea* produces an immunosuppressive factor and degrades immunoglobulins^45, 46^. These results highlight the potential relationship among the tonsil environment, tonsillar microbiota, and clinical features and suggest that the tonsillar microbiota contributes to kidney diseases, including IgAN.

Our study had several limitations. First, the number of subjects was relatively small. Considering the high variability in microbiota composition, caution is needed when applying these results to the general population. Second, there was an age difference between the healthy control group and other groups. Though we showed that there was no obvious difference in bacterial diversity according to age, age is still a powerful parameter and may affect the composition of the tonsillar microbiota. Age- and sex-matched subjects should be compared in a future study. Third, we obtained samples by tonsillar swabs, which may only reflect superficial bacteria. There was also a lack of negative sequencing controls to check potential contamination of sequencing reagents, however we evaluated potential contamination at every experimental step using negative controls (distilled water) and empty swabs. Despite these limitations, our results provide key insights into the tonsillar microbiota in IgAN, including correlations between taxon abundances and clinical features.

In conclusion, the microbiota in the tonsils of patients with IgAN differed from those of patients with other kidney diseases and healthy controls. The high relative abundances of *Rahnella*, *Ruminococcus_g2*, and *Clostridium_g21* in patients with IgAN could be related to the immune status and pathogenesis of the disease. Further studies with larger sample sizes and systemic analyses of the gut microbiota and immunological features are necessary to understand the role of the microbiota in IgAN development.

## Methods

### Study subjects and sample collection

Patients who were admitted to undergo a kidney biopsy at three medical centres (Seoul National University Hospital, Seoul National University Boramae Medical Center, and Kangwon National University Hospital) in South Korea were enrolled. Patients were assigned to IgAN, DN, and MN groups based on the pathological evaluation. The DN and MN groups were used as disease control groups. Participants were enrolled at the time of kidney biopsy, before treatment with steroids or other immunosuppressants. Subjects who visited the Seoul National Boramae Medical Center for a regular health check-up and had normal kidney function and no underlying disease served as healthy controls. All subjects were over 18 years-old, and subjects who had undergone tonsillectomy were excluded. The demographic and clinical data for subjects were collected from hospital electronic medical records. The eGFR was calculated using the Chronic Kidney Disease-Epidemiology Collaboration equation^47^. A histological evaluation was performed according to the Oxford classification^48^. Tonsil swab samples were collected by rubbing each palatine tonsil twice using a cotton swab (Easy Swab; Synergy Innovation, Seongnam, South Korea). To check a potential contamination in cotton swab, empty swabs were also analysed along with tonsil swab samples. The samples were immediately stored at 4 °C, delivered to the laboratory within 24 h, and then stored at − 80 °C until DNA extraction.

This study was approved by the Institutional Review Board of each centre (Seoul National University Hospital IRB No. 1508-046-694, Seoul National University Boramae Medical Center IRB No. 26-2015-128, Kangwon National University Hospital IRB No. KNUH-2015-07-003) and performed in accordance with the principles of the Declaration of Helsinki. Informed written consent was obtained from each subject.

### DNA extraction and MiSeq sequencing

Metagenomic DNA was extracted from 80 swab specimens using a FastDNA SPIN Extraction Kit (MP Biomedicals, Santa Ana, CA, USA) according to the manufacturer’s instructions. For MiSeq sequencing, samples were prepared as described previously^49, 50^. Briefly, the V4-5 variable region of the 16S rRNA gene was amplified using extracted DNA, and amplification was performed according to the protocol for preparing a 16S metagenomics sequencing library using the MiSeq system (Illumina, Inc., San Diego, CA, USA). The first step of amplification was performed in a final volume of 50 µl containing 1 µM of each primer, 2.5 U Ex Taq polymerase (Takara Bio, Otsu, Japan), 5 µl of 10 × Ex Taq buffer, 4 µl dNTP mixture, and 2 µl template DNA using a C1000 Touch thermal cycler (Bio-Rad, Hercules, CA, USA) under the following conditions: initial denaturation at 95 °C for 3 min; 25 cycles of denaturation at 95 °C for 30 s, annealing at 55 °C for 30 s, and extension at 72 °C for 30 s; and final extension at 72 °C for 5 min. The purification and size selection of amplicon were performed using Agencourt AMPure XP beads (Beckman Coulter, Indianapolis, IN, USA). The index PCR was performed using 5 µl of purified PCR product in a final volume of 50 µl using the Nextera XT Index Kit (Illumina) under the following conditions: initial denaturation at 95 °C for 3 min; 8 cycles of denaturation at 95 °C for 30 s, annealing at 55 °C for 30 s, and extension at 72 °C for 30 s; and final extension at 72 °C for 5 min. Purification and size selection were performed again using Agencourt AMPure XP Beads (Beckman Coulter). Negative controls (distilled water) were used at every step to check contamination, and the same experiments were performed for swab contamination controls. No amplicons were detected in negative controls and swab contamination controls. The quantification of library was performed using a PicoGreen dsDNA Assay Kit (Invitrogen, Calsbad, CA, USA). Equimolar concentrations of each library were pooled and sequenced on the Illumina MiSeq System (250-bp paired-end reads) according to the manufacturer’s instructions.

### Sequence data analysis

Sequence reads were analysed using CLC genomics workbench v.11.0.1 with the Microbial Genomic Module (Qiagen, Aarhus, Denmark) as described previously^49, 51^. Sequence reads were merged, and reads with short lengths (merged reads of < 200 bp) or low-quality scores (Q < 25) and primer sequences were removed from the merged sequences using the USEARCH pipeline v.10.0.240 (https://www.drive5.com/usearch). Chimeric sequences were removed using the UPARSE tool. Resultant sequences were clustered into OTUs based on97% identity. Taxonomic positions of representative sequences in each OTU cluster were assigned using the EzTaxon-e database^52^. To compare diversity indices among samples, read numbers were normalised by random subsampling and indices were calculated using MOTHUR^53^. PCoA plots were generated to compare the microbiota among samples using Calypso^54^.

### Statistical analyses

Clinical characteristics of subjects were compared by analysis of variance (ANOVA) and chi-square tests. P-values < 0.05 were considered statistically significant. Permutation tests were used to calculate statistical significance in the PCoA. Differences in microbial taxa between samples were evaluated by the Mann–Whitney U test and Kruskal–Wallis test implemented in R. The correlations between clinical features and relative abundances of specific microbes were analysed using Spearman’s rank correlation tests in SPSS (version 22). Corrections for multiple testing were performed using the Benjamini–Hochberg method for false discovery rate adjustment. Results with corrected p-values of < 0.05 were considered statistically significant.

## Supplementary information

Supplementary file1

Supplementary file2

Supplementary file3

Supplementary file4

Supplementary file5

Supplementary file6

Supplementary file7

## Data Availability

All sequences were deposited on European Nucleotide Archive (ENA) study accession number PRJEB39311 (https://www.ebi.ac.uk/ena/data/view/PRJEB39311).
