# Supplementary figures and images for: Comparative analysis of the tonsillar microbiota in IgA nephropathy and other glomerular diseases
Source: Sci Rep. 2020 Oct 1;10:16206. doi: 10.1038/s41598-020-73035-x (PMC7530979; doi:10.1038/s41598-020-73035-x)

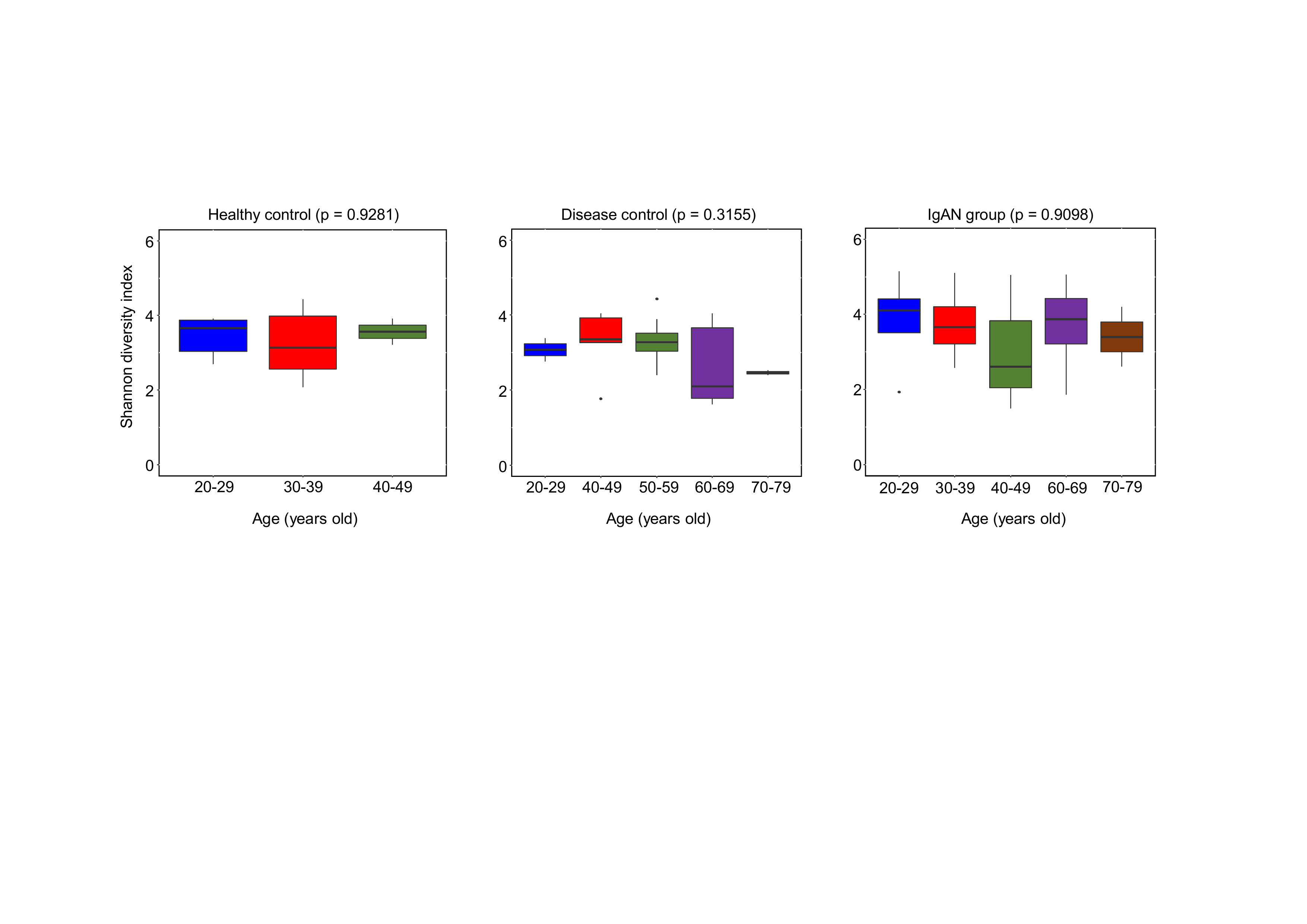

Supplement: Supplementary file 1 — Supplementary file1 [file 41598_2020_73035_MOESM1_ESM.tif]

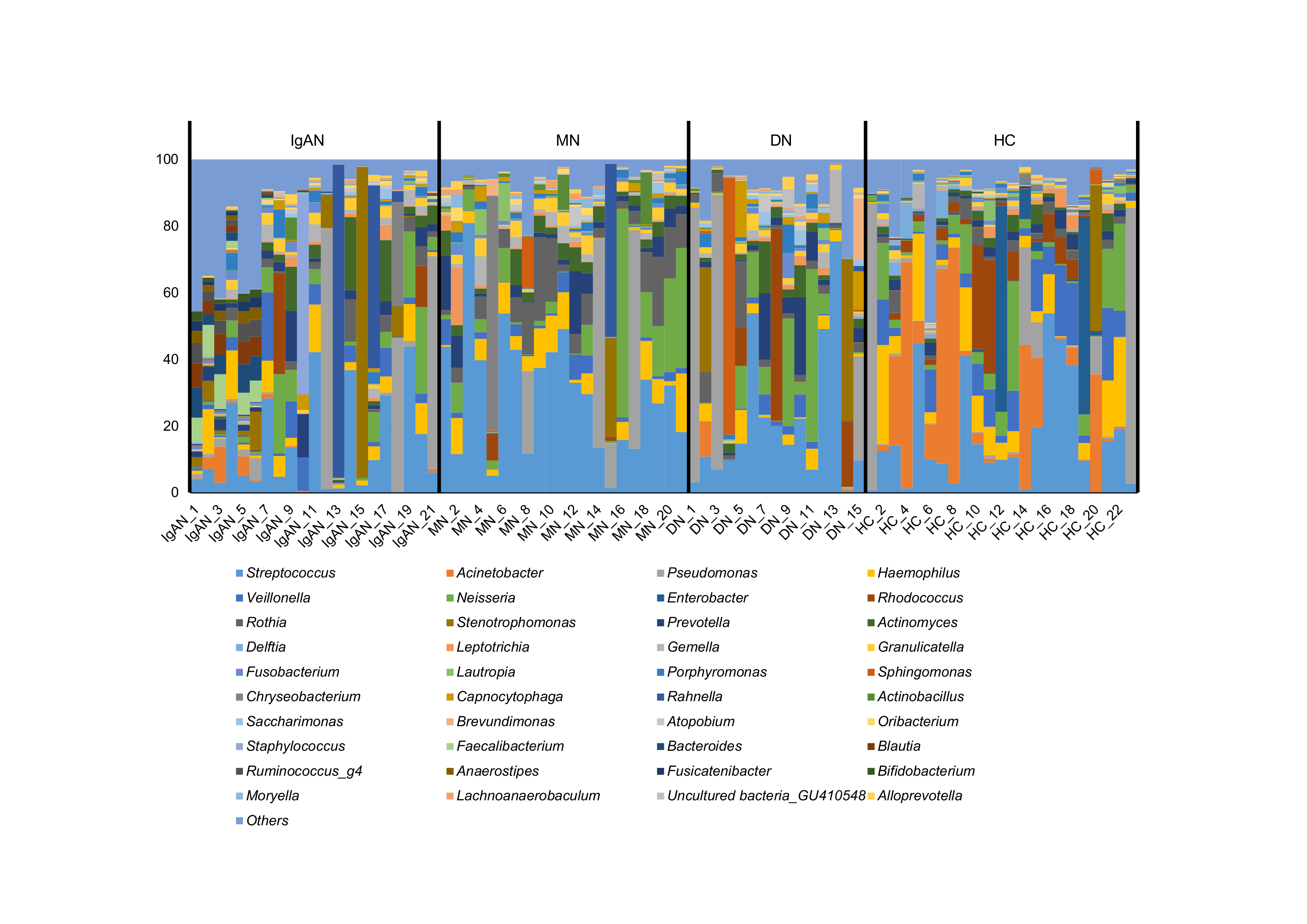

Supplement: Supplementary file 2 — Supplementary file2 [file 41598_2020_73035_MOESM2_ESM.tif]

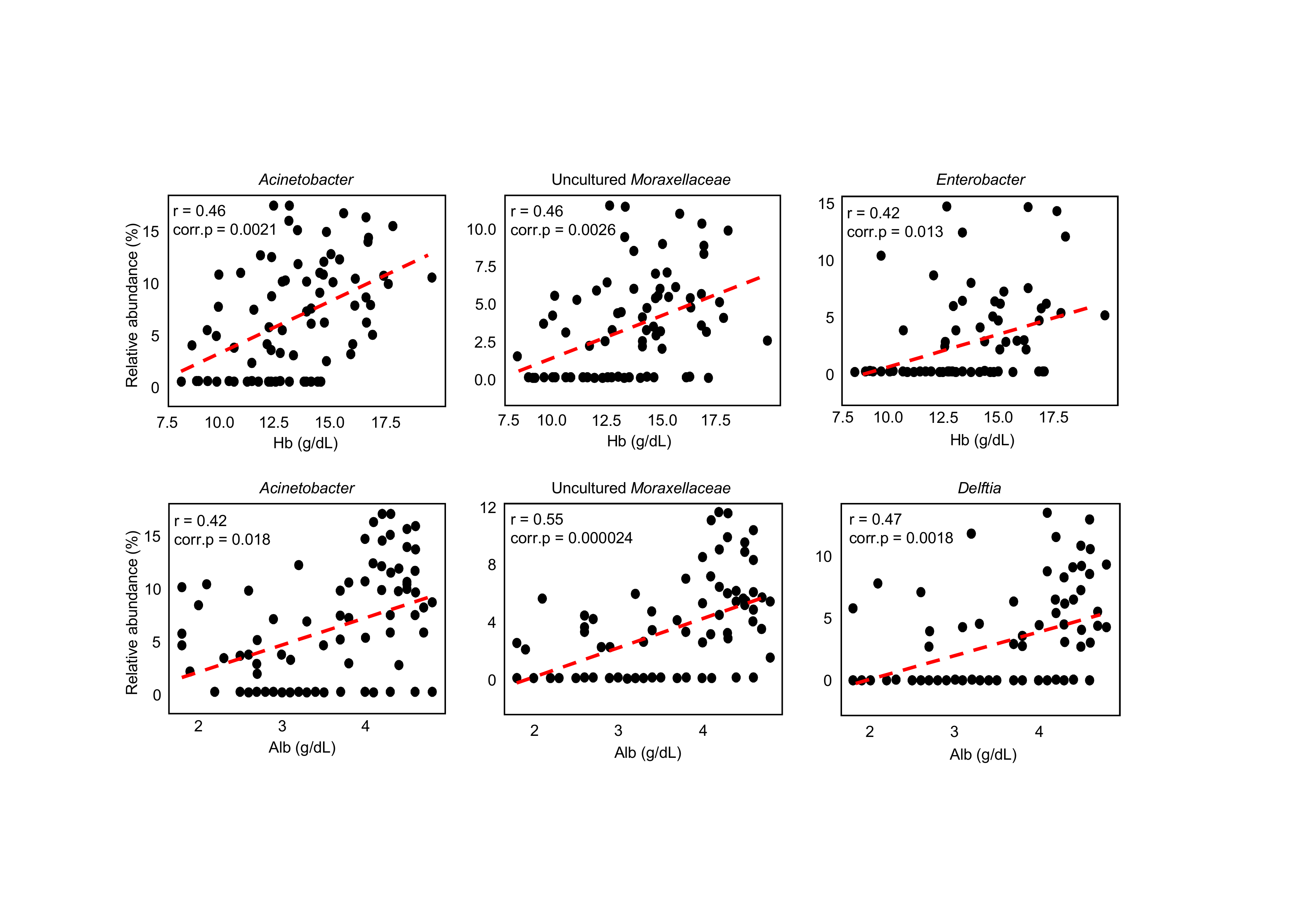

Supplement: Supplementary file 3 — Supplementary file3 [file 41598_2020_73035_MOESM3_ESM.tif]

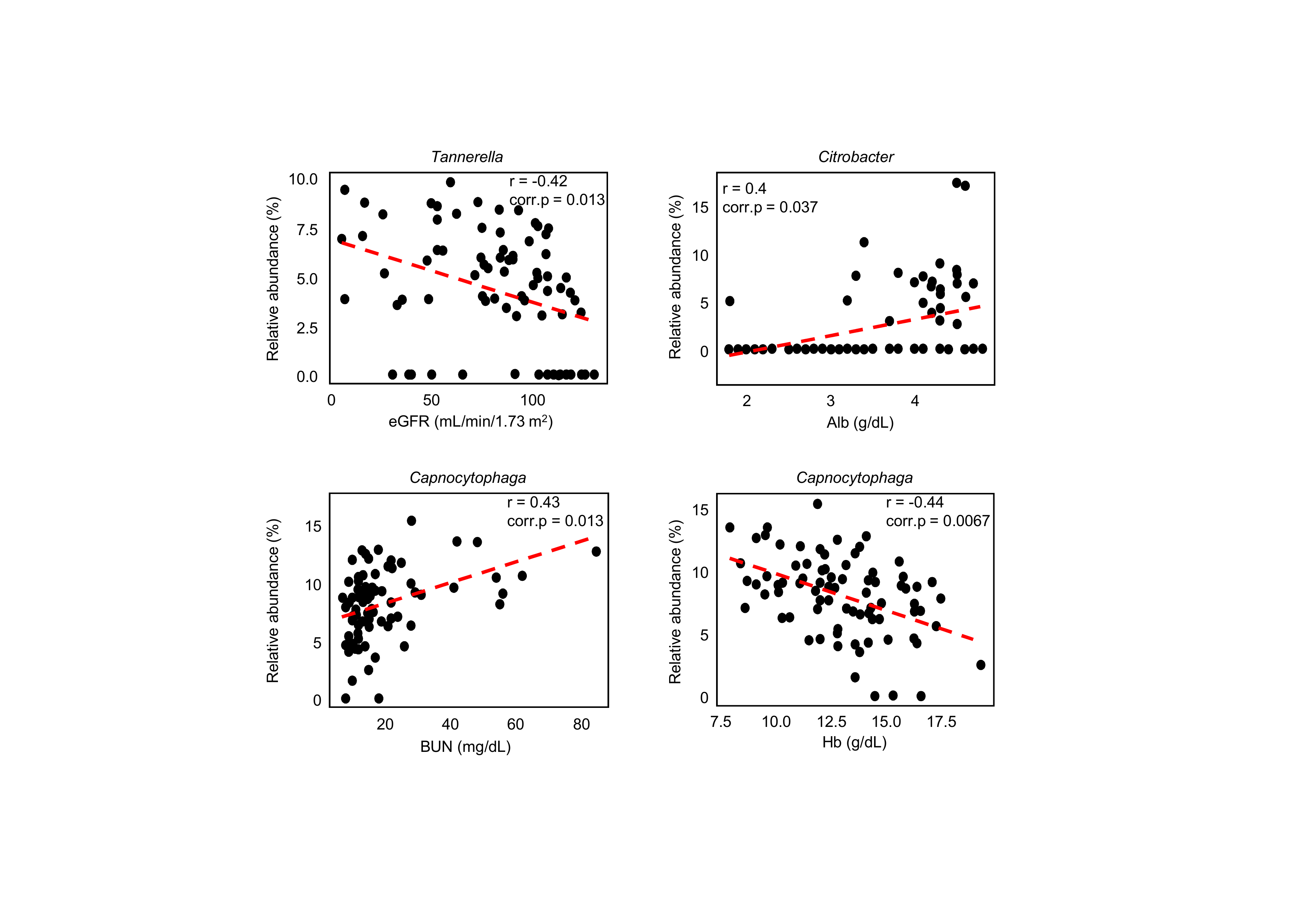

Supplement: Supplementary file 4 — Supplementary file4 [file 41598_2020_73035_MOESM4_ESM.tif]
