# Supplementary material for: Comparative analysis of the tonsillar microbiota in IgA nephropathy and other glomerular diseases
Source: Sci Rep. 2020 Oct 1;10:16206. doi: 10.1038/s41598-020-73035-x (PMC7530979; doi:10.1038/s41598-020-73035-x)
Supplement: Supplementary file 5 — Supplementary file5 [file 41598_2020_73035_MOESM5_ESM.pdf]

TITLE: Comparative analysis of the tonsillar microbiota in IgA nephropathy and other glomerular diseases

AUTHOR'S NAME: Ji In Park, Tae-Yoon Kim, Bumjo Oh, Hyunjeong Cho, Ji Eun Kim, Seong Ho Yoo, Jung Pyo Lee, Yon Su Kim, Jongsik Chun, Bong-Soo Kim, Hajeong Lee

Supplementary information

Supplementary Table S1. Summary of diversity indices based on normalised reads.

Supplementary Table S2. Genera with significant differences in abundance between groups. IgAN: Immunoglobulin A nephropathy, MN: Membranous nephropathy, DN: Diabetic nephropathy, HC: Healthy control.

Supplementary Figure S1. Comparison of bacterial diversity according to age within each group.

Supplementary Figure S2. Composition of genera in the tonsillar microbiota from all samples.

Supplementary Figure S3. Correlations between tonsillar microbes and clinical features. Genera differed significantly between patients with IgAN and healthy controls. The correlations were selected by corrected p-values.

Supplementary Figure S4. Correlations between tonsillar microbes and clinical features. Genera were significantly different between healthy controls or patients with IgAN and other kidney diseases. The correlations were evaluated by corrected p-values.
